# EYA1 expression and subcellular localization in neuroblastoma and its association with prognostic markers

**DOI:** 10.14312/2052-4994.2016-3

**Published:** 2016-05-05

**Authors:** Jeanne N. Hansen, Louis T. Lotta, Allison Eberhardt, Nina F. Schor, Xingguo Li

**Affiliations:** 1 Department of Pediatrics, University of Rochester School of Medicine and Dentistry, Rochester, NY, USA

**Keywords:** neural crest tumors, transcription factors, biomarkers, prognosis

## Abstract

Neuroblastoma, the most frequently occurring extracranial solid tumor of childhood, arises from neural crest-derived cells that are arrested at an early stage of differentiation in the developing sympathetic nervous system. There is an urgent need to identify clinically relevant biomarkers for better prognosis and treatment of this aggressive malignancy. Eyes Absent 1 (EYA1) is an essential transcriptional coactivator for neuronal developmental programs during organogenesis. Whether or not EYA1 is implicated in neuroblastoma and subcellular localization of EYA1 is relevant to clinical behaviour of neuroblastoma is not known. We studied EYA1 expression and subcellular localization by immunohistochemistry in tissue microarrays containing tumor specimens from 98 patients, 66 of which were characterized by known clinical prognostic markers of neuroblastoma. Immunostaining results were evaluated and statistically correlated with the degree of histologic differentiation and with neuroblastoma risk stratification group characteristics, including stage of disease, patient age, tumor histology and mitosis–karyorrhexis index (MKI), respectively. We found that EYA1 levels were significantly higher in neuroblastomas than in ganglioneuromas and ganglioneuroblastomas. EYA1 was more highly expressed in stage 1,2,3 or 4S tumors as compared to stage 4 tumors (P<0.01). Tumors with high levels of nuclear EYA1 were more frequently associated with high nuclear MYCN levels. These results suggest that modulation of expression and intracellular localization of EYA1 in neural crest cells may provide a novel direction for therapeutic strategies.

## Introduction

Neuroblastoma, the most common extracranial tumor of childhood, accounts for 15% of childhood cancer deaths. Despite improvements in treatment during the past several decades, prognoses remain dire for patients with high-risk disease, which account for nearly half of children with neuroblastoma. A large proportion of these cases are associated with treatment-resistant tumors and frequent relapses, with a 5-year overall survival rate ranging from 20% to 50% [1]. The underlying oncogenic mechanisms of this aggressive malignancy are largely unknown. Aberrant expression of MYCN is strongly associated with poor outcome, making this oncoprotein one of few proposed targets in molecular therapy for neuroblastoma [1]. Similar to other transcription factors, MYCN is technically challenging to target pharmacologically. Thus, novel molecular markers of tumor aggressiveness and targets for therapy are urgently needed to improve prognosis and treatment of this devastating disease.

Eyes Absent (EYA), originally identified in Drosophila and essential for eye development, plays significant roles in organogenesis by controlling cell proliferation, differentiation and survival [2]. Mechanistically, these functions are associated with a unique combination of EYA biochemical activities: phosphatase activity and transactivation potential when associated with DNA-binding partners. Additionally, aberrant activity of human homologs of EYA (EYA1–4) have been linked to oncogenesis in a variety of cancers [3]. For example, EYA1 is overexpressed in Wilms tumor [4], breast cancer [5], malignant glioma [6] and medulloblastoma [7] but down-regulated in gastric cancer [8]. Overexpression of EYA2 has been observed in epithelial ovarian cancer [9], lung adenocarcinoma [10] and breast cancer [11]; silencing of EYA2 gene expression has been observed in colorectal cancer [12] and pancreatic cancer [13]. EYA3 is overexpressed in Ewing sarcoma [14]. EYA4 is overexpressed in esophageal adenocarcinoma [15], colorectal cancer [16] and malignant peripheral nerve sheath tumor [17]. All together these observations suggest that the oncogenic or anti-oncogenic roles of misregulated EYA expression may be context-dependent.

Since EYA1 has a known role in neurogenesis during development [18] and EYA homologs, including EYA1, have recently been linked to other paediatric malignancies [7,14], we investigated the expression of EYA1 in neuroblastoma. We utilized immunohistochemistry to examine the expression patterns of EYA1 in tissue microarrays (TMAs) composed of tissue samples collected from 98 individual patients, including 66 cases of neuroblastoma, and looked for correlation with standard-risk determining diagnostic factors. Our results show that EYA1-postive neuroblastoma tumors were more likely diagnosed in children with stage 1,2,3 and 4S disease. Moreover, we identified a trend towards association of nuclear EYA1 with nuclear MYCN levels in aggressive tumors. We conclude that EYA1 may be a potential biomarker for neuroblastoma prognosis, and modulation of its expression and subcellular localization in neural crest cells may contribute to the therapy of neuroblastoma.

## Materials and methods

### Neuroblastoma TMAs

Neuroblastoma TMAs were obtained from the Children’s Oncology Group (COG) Biospecimen Repository (Nationwide Children’s Hospital, Columbus, OH). Normal tonsil tissue as well as ganglioneuroma/ganglioneuroblastoma tumor tissue samples were included in the TMAs as control tissue.

### Analyses of patient mRNA expression data

The publicly available Versteeg [19] and Kocak [20] microarray gene expression datasets were obtained from the R2 microarray analysis and visualization platform (http://hgserver1.amc.nl/cgi-bin/r2/main.cgi?&species=hs). Kaplan-Meier analysis was conducted online, and the resulting survival curves and P values (log-rank test) were downloaded. All cutoff values for separating high and low expression groups were determined by the online algorithm.

### Cell culture, transfection and coimmunoprecipitation (coIP) analysis

KELLY cells were purchased from Sigma (St. Louis, MO) and cultured in DMEM:F12, supplemented with 10% FBS. HEK293TN cells were cultured in DMEM with 10% FBS. Transient transfection was performed by using jetPRIME (Polyplus transfection, #114-07) per the manufacturer’s instructions. CoIP analysis was performed essentially as previously described[21]. Briefly, two days post-transfection, whole cell lysates of HEK293TN cells were prepared and used for V5-IP (Invitrogen, #R960-25). In the case of KELLY cells, nuclear extracts were isolated and subjected to immunoprecipitation with anti-MYCN antibody (Santa Cruz, #sc-53993), followed by immunoblotting with anti-EYA1 antibody (Prosci, #25-067). The following antibodies were used in western blot: *α*-Flag (Thermo Fisher Scientific, #MA1-91878) and *β*-actin (Sigma, #A5316).

### Immunohistochemistry

Immunohistochemical studies were performed essentially as previously described [22]. Briefly, prior to immunostaining tissue underwent deparaffinization in xylene, followed by antigen retrieval in citrate buffer, and blocking of endogenous peroxidase. The immunohistochemical staining was done using a standard labelled streptavidin-biotin method (VECTASTAINVR ABC Kit, # PK-4001, Vector Laboratories, CA, USA) followed by 3,3′-diaminobenzidine enzymatic development. The nonspecific binding was blocked by incubating with goat serum and Avidin D/Biotin. The sections were incubated with anti-EYA1 antibody (Prosci, #25-067, 1:100 dilution) or anti-MYCN antibody (Santa Cruz, #sc-53993, 1:100 dilution) for 30 min and then goat anti-rabbit or anti-mouse biotinylated IgG for 30 min, followed by incubation with ABC reagents. Hematoxylin was used for nuclear counterstaining. Slides were dehydrated through graded alcohol and xylene washing, and mounted on cover slips. Normal rabbit or mouse IgG served as the negative control. Slides were imaged with an Olympus VS120 slide scanner system.

### Scoring

Immunoreactivity for EYA1 was independently scored by two investigators (JH and LL), both blinded as to the identity and risk group of each specimen. The results were classified according to fractions of positive cells and general intensity of positive cells: 0 (negative), <10% positive cells or negative staining; 1 (low), 10–50% positive cells with weak or moderate intensity or 10–25% positive cells with strong intensity; 2 (high), >50% positive cells or 25–50% positive cells with strong intensity. Cytoplasmic staining was defined by majority of cells with cytoplasmic localization only. Nuclear staining was defined as majority of cells that have either nuclear or both nuclear and cytoplasmic staining. The consensus was high between the two scorers and conflicting results between scorers were re-scored by both and a consensus was reached.

### Statistical analysis

Prevalence of neuroblastoma cells that contain EYA1 was individually correlated with neuroblastoma risk stratification group [including stage of disease, age, tumor histology and Mitosis–Karyorrhexis Index (MKI)] of 66 individual patients for whom we had partial sets of risk-determining information. The association of EYA1 protein expression with these prognostic factors was examined by a two-tailed Fisher’s exact test with a 95% confidence interval; *P* values <0.05 were considered significant.

## Results

### Relationship of relative EYA1 mRNA and protein levels to degree of histologic differentiation of neural crest tumors

**Figure 1:**
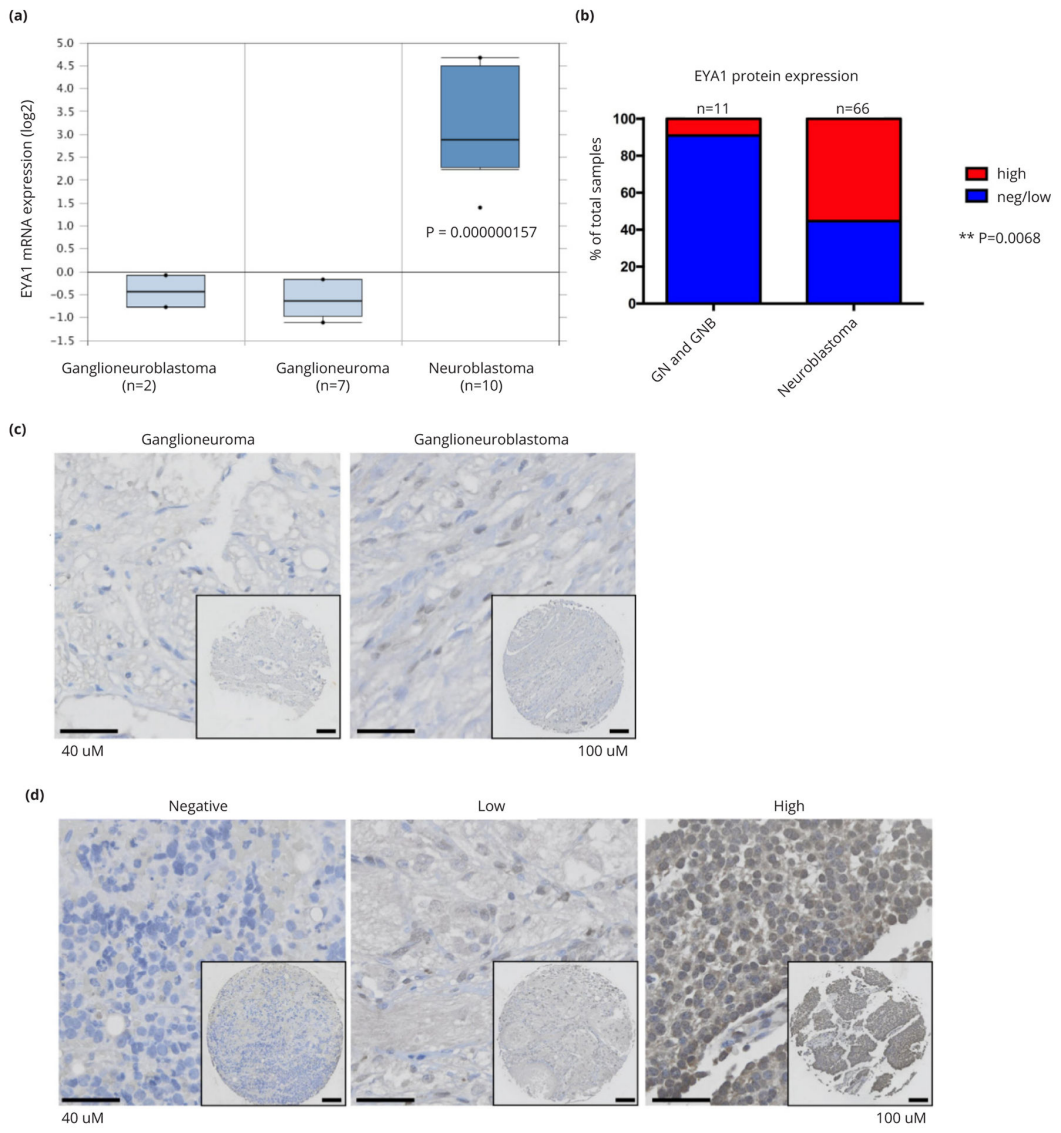
EYA1 mRNA and protein expression is significantly associated with more primitive points in the neural crest lineage. (a) EYA1 is highly expressed in neuroblastomas compared with ganglioneuroblastoma and ganglioneuroma. The graph was downloaded from Oncomine microarray database (www.oncomine.com). (b) EYA1 protein expression in ganglioneuroma/ganglioneuroblastoma and neuroblastoma patients. **: P<0.01. Immunostaining for EYA1 in ganglioneuroma/ganglioneuroblastoma (c) and neuroblastoma (d).

Peripheral neuroblastic tumors include ganglioneuroma, ganglioneuroblastoma and neuroblastoma. Ganglioneuromas consist of differentiated, stroma-rich ganglion cells and are classified as low risk, while neuroblastomas are undifferentiated, stroma-poor and high-risk. Ganglioneuroblastomas are of variable risk, and, in accordance with this, consist of intermixed populations of cells of variably differentiated histology. Using the Oncomine cancer microarray database (www.oncomine.com), we found that EYA1 mRNA levels were significantly higher in neuroblastoma tumors than in ganglioneuroma and ganglioneuroblastoma (Figure 1a). To extend our studies to protein levels of EYA1, we stained neuroblastoma TMAs for EYA1. As exemplified in Figure 1, immunostaining for EYA1 in ganglioneuroma or ganglioneuroblastoma was generally lower (1b and 1c) than was the case for neuroblastoma (P=0.0068; Figure 1b and 1d). In this series of 66 neuroblastomas, 41 showed a high-level of EYA1 protein expression and 25 showed negative or low EYA1 expression. In contrast, all of the ganglioneuromas (n=3) and 7 of 8 ganglioneuroblastomas showed negative or low EYA1 protein expression, while one ganglioneuroblastoma showed high EYA protein expression. This inverse correlation of EYA1 expression with degree of differentiation of neural crest tumors is in keeping with the developmental expression and critical role in cell survival of EYA1 during early neurogenesis, but not once neural or glial differentiation has occurred [2].

**Figure 2:**
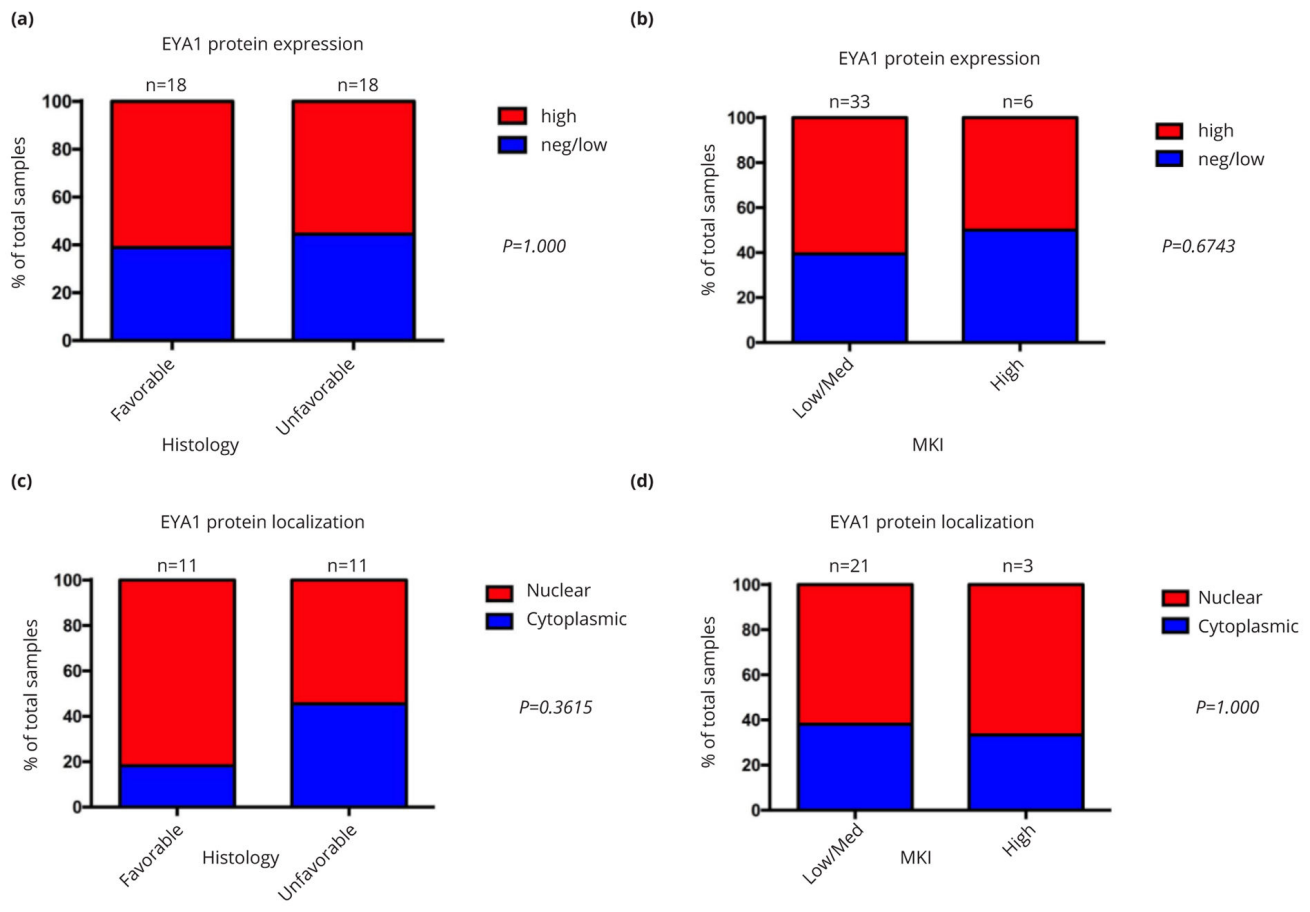
Relationship of EYA1 protein expression and intracellular localization to histologic differentiation of neuroblastoma. Bar graphs show case distribution of EYA1 total levels and subcellular localization by histology (a, c) and MKI (b, d).

To determine the relationship of differentiation and, as a surrogate for it, proliferation index to EYA1 expression in neuroblastomas, we examined EYA1 protein immunostaining as a function of favourable versus unfavourable histology and MKI, respectively. As is shown in Figure 2a and 2b, despite the relationship between EYA1 content and neural crest tumor major differentiation class, among neuroblastomas, there was no statistically significant difference in EYA1 protein level between tumors with favourable or unfavourable histology, or between tumors with low or intermediate MKI (i.e., ≤ 4%) and those with high MKI.

### Relationship of EYA1 intracellular localization to degree of histologic differentiation of neural crest tumors

EYA shows both cytoplasmic and nuclear localization. Once in the nucleus, EYA may function in the DNA damage response or as a transcriptional coactivator [2]. As such, its intracellular activity depends critically upon its translocation from the cytoplasm to the nucleus. We therefore examined the relationship between favourable versus unfavourable histology and MKI, respectively, and the intracellular localization of EYA1 (Figure 2c and 2d). There is an association of nuclear localization of EYA1 with higher MKI. This is also consistent with the predominantly cytoplasmic localization of EYA1 in non- and less malignant ganglioneuromas and ganglioneuroblastomas (Figure 1c).

### Relationship of EYA1 protein levels and intracellular localization to patient age and disease stage in neuroblastoma

**Figure 3:**
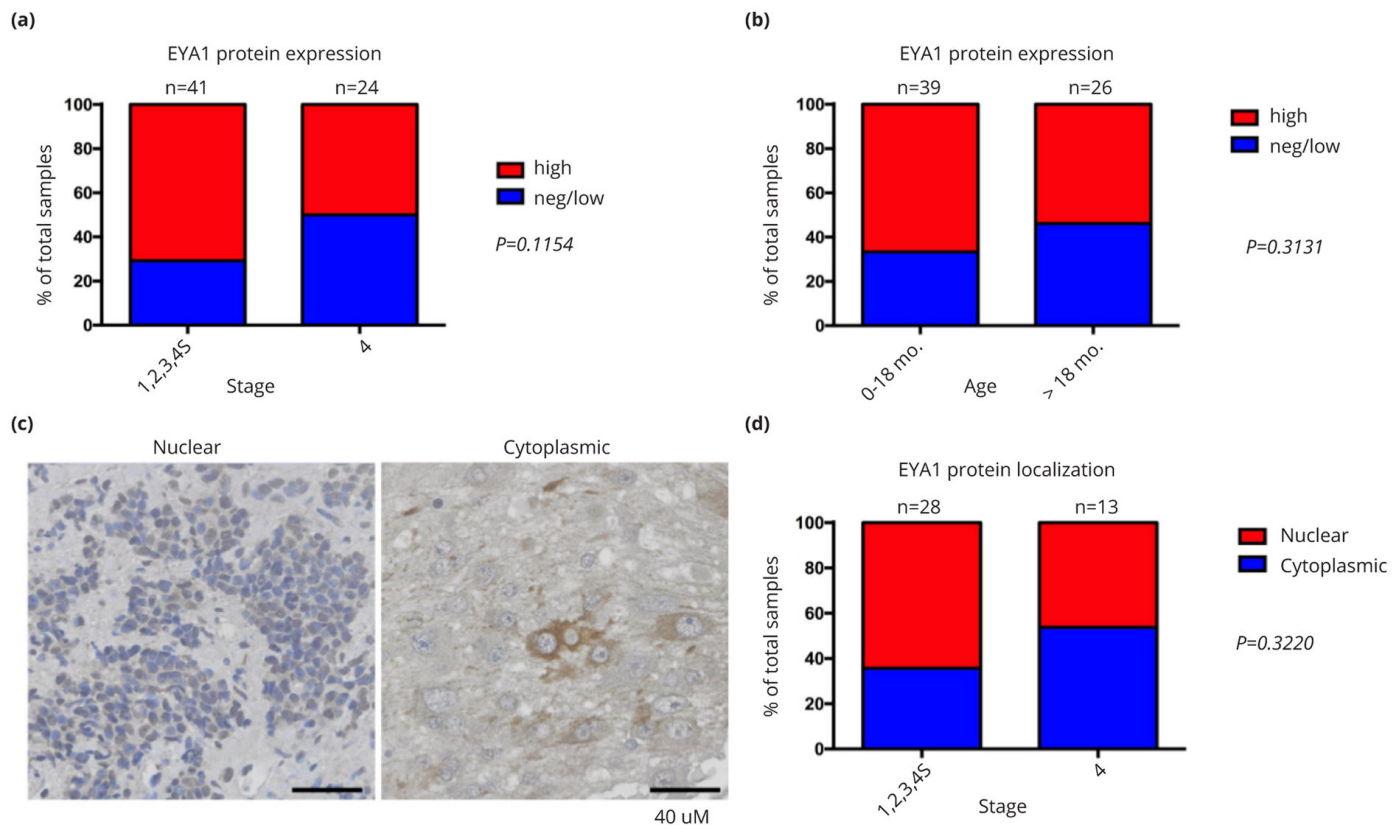
Relationship of EYA1 protein expression and intracellular localization to patient age and disease stage in neuroblastoma. Bar graphs show case distribution of EYA1 total levels by INSS stage (a) and age at diagnosis (b). (c) Representative images of immunostaining for EYA1 subcellular localization, (d) Bar graph shows case distribution of EYA1 subcellular localization by INSS stage.

As shown in Figure 3a, EYA1 staining correlates inversely with advanced neuroblastoma stage. Stage 1, 2, 3 and 4S disease is associated with higher EYA1 protein expression than stage 4 disease (P=0.1154). Moreover, there is a strong trend toward a correlation between diagnosis at early age (≤18 months) and high expression of EYA1 protein (relative to values for age >18 months at diagnosis; Figure 3b).

To test the hypothesis that nuclear EYA1 predominates in advanced stage neuroblastoma, we scored the TMA specimens with high EYA1 expression (41 in total) for cytoplasmic and nuclear EYA1 localization (Figure 3c). Since only half of 24 stage 4 tumors can be scored for cytoplasmic/ nuclear localization, the small size of the cohort does not provide adequate power for statistical analysis (Figure 3d). But the potential association between nuclear localization of EYA1 and advanced stage of neuroblastomas will be followed up in future studies to understand and confirm this relationship.

### Correlation between EYA1 nuclear localization and high MYCN protein levels in neuroblastomas

**Figure 4:**
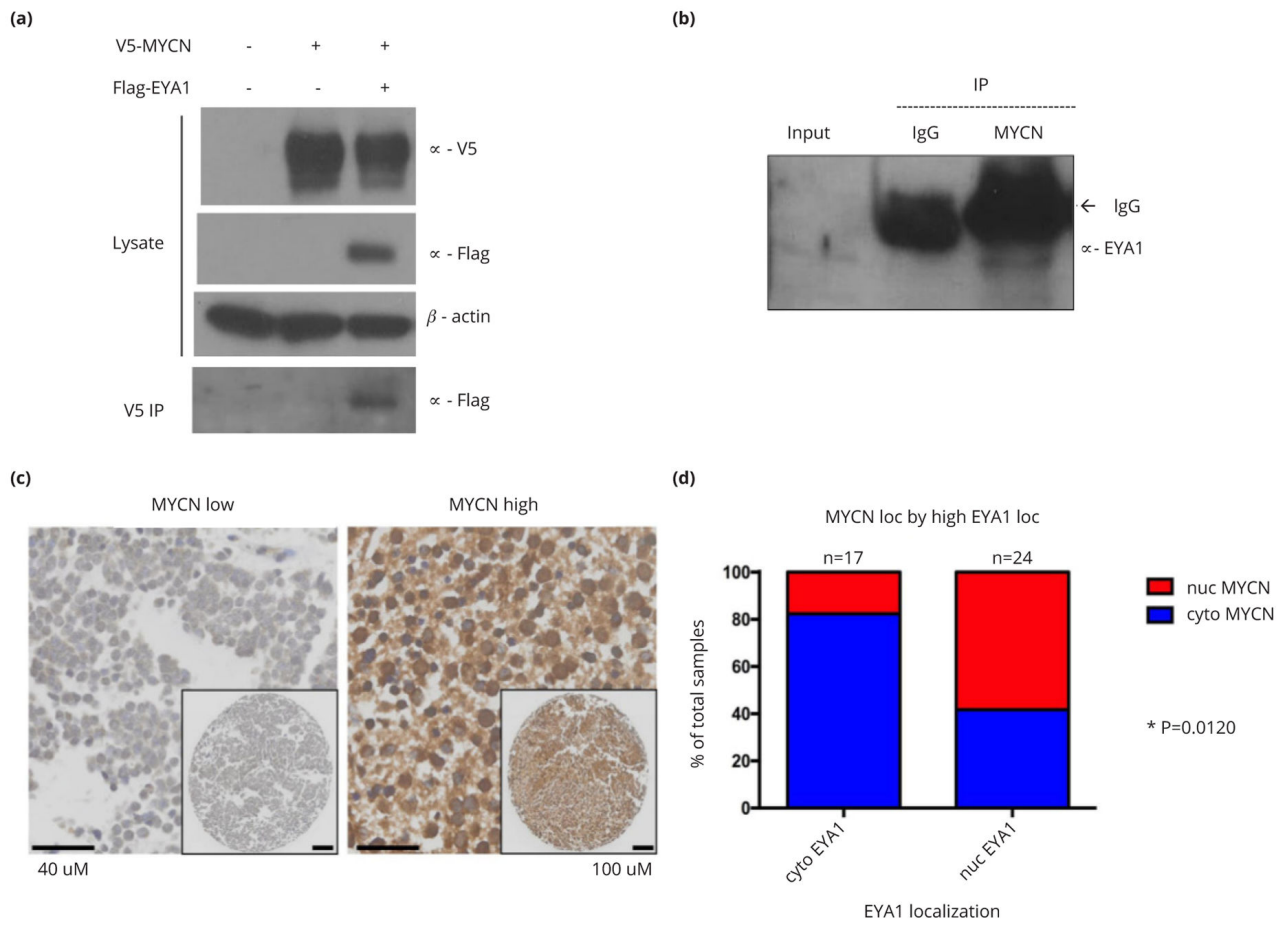
EYA1 interacts with MYCN and nuclear localization of EYA1 correlates with high nuclear MYCN levels in neuroblastoma. (a) V5-tagged MYCN and Flag-tagged EYA1 were co-transfected into HEK293TN cells and whole cell lysate were subjected to IP with *α*-V5 antibody, followed by western blot with *α*-Flag antibody. *β*-actin was used as a loading control. (b) Endogenous MYCN physically interacts with EYA1. Nuclear extract from KELLY cells were subjected to CoIP analysis with *α*-MYCN antibody, followed by immunoblotting with *α*-EYA1 antibody. (c) Representative images of immunostaining for MYCN in primary neuroblastoma tumors. (d) Bar graph shows case distribution of MYCN subcellular localization in high EYA1-expressiong tumors with cytoplasmic or nuclear localization. *: P<0.05.

MYCN was recently identified as an EYA1 binding factor through a yeast two-hybrid screen from a mouse cDNA library and EYA1 was shown to stabilize MYCN protein via regulation of phosphorylation [23]. However, the relationship between EYA1 and MYCN in the context of neuroblastoma has not been explored. We first confirmed the physical interaction between EYA1 and MYCN in human embryonic kidney cell line HEK293TN cells upon cotransfection of V5-tagged MYCN and Flag-tagged EYA1 (Figure 4a). We then examined whether endogenous *MYCN* forms a complex with EYA1 in neuroblastoma cells. CoIP analysis of nuclear extract from KELLY cells carrying MYCN amplification demonstrated a physical association between endogenous MYCN and EYA1 proteins (Figure 4b). We then tested the hypothesis that nuclear EYA1 levels are correlated with MYCN levels in primary neuroblastoma tumor specimens. We found a significant association between high EYA1 expression with nuclear localization and high nuclear MYCN protein levels (P=0.0120; Figure 4c and 4d). This observation further suggests that EYA1 and MYCN may function together during neuroblastoma tumor proliferation. While further studies are needed to confirm this observation, these results suggest a mechanistic basis for the prevalence of EYA1 nuclear localization in advanced neuroblastoma tumors.

### Relationship between EYA1 mRNA expression and overall survival in neuroblastoma patients

**Figure 5:**
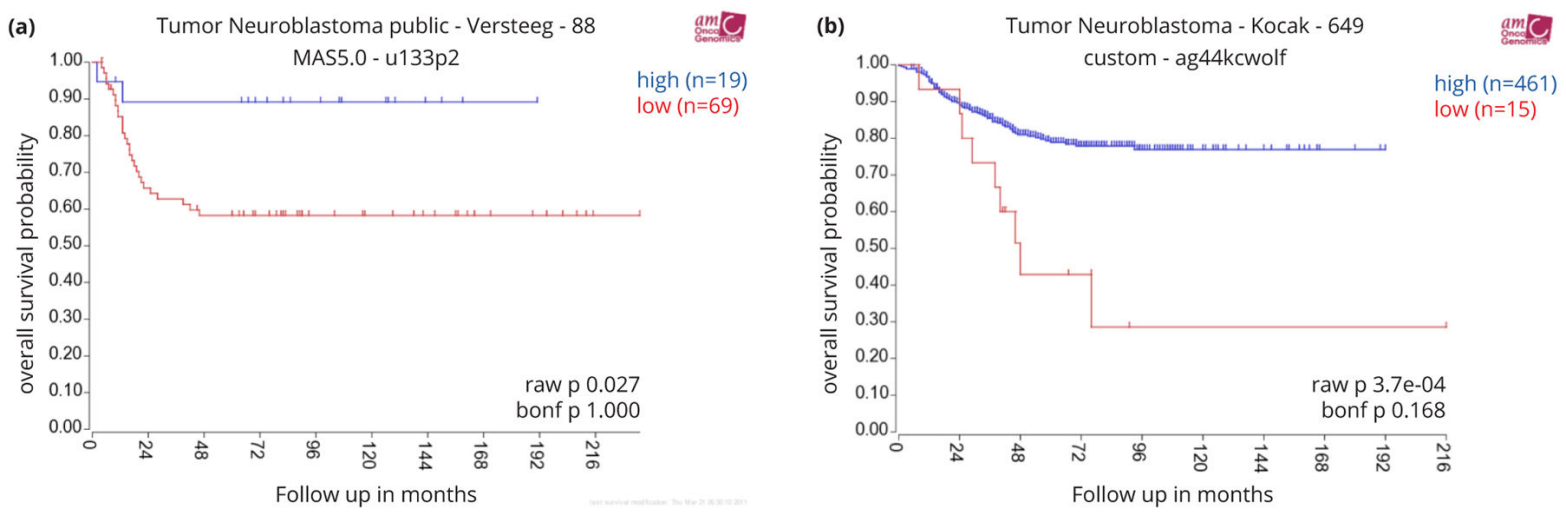
Figure 5a,b High EYA1 mRNA levels are significantly associated with good prognosis in neuroblastoma patients. Kaplan-Meier survival plots were downloaded from R2 microarray analysis and visualization platform (http://hgserver1.amc.nl/cgi-bin/r2/main.cgi?&species=hs). Patients with higher EYA1 expression are highlighted in blue, whereas patients with lower EYA1 expression are highlighted in red. The log-rank test P values are indicated.

Due to the small sample size and incomplete clinical information that precluded statistical power for analysis, we were unable to determine directly from our TMA cohort the clinical relevance of nuclear EYA1. Therefore we then investigated the relationship of EYA1 mRNA expression to overall survival in neuroblastoma patients by performing Kaplan-Meier analysis of survival for the publicly available neuroblastoma datasets. As shown in Figure 5a, high EYA1 mRNA levels were associated with good prognosis, whereas low EYA1 expression was associated with poor outcome in the Versteeg dataset consisting of a cohort of 88 neuroblastoma patients. We confirmed that high EYA1 mRNA levels are prognostic for favorable outcome with the Kocak dataset (Figure 5b), which includes a cohort of 476 neuroblastoma patients. Taken together, our analyses of 2 independent mRNA microarray datasets and the Children’s Oncology Group TMAs suggest EYA1 as a novel prognostic marker in neuroblastoma.

## Discussion

This study provides several lines of evidence for a relationship between EYA1 expression and the biology and clinical behavior of neuroblastoma. First, cellular EYA1 protein expression in neuroblastomas is associated with low disease stage and young patient age at diagnosis (detailed data and analysis presented in Table 1), both prognostic factors for good outcome. Second, high EYA1 mRNA levels are prognostic for favorable outcome in 2 independent gene expression datasets that collectively examine tumors from 564 neuroblastoma patients. In support of this model, we also found high EYA1 protein levels in stage 4S neuroblastoma specimens, a stage of disease associated, by definition, with young infants, and uniquely at high likelihood of spontaneous regression. These results suggest that EYA1 may represent a novel prognostic marker for neuroblastoma. While, at first, the absence of expression of EYA1 in ganglioneuromas might seem paradoxical, EYA1 protein is efficiently degraded during normal development at the point of M-to-G1 transition [24].

EYA is a component of a highly conserved regulatory network that plays an essential role during organogenesis in organisms ranging from insects to humans. Vertebrates encode 4 EYA genes (EYA1–4) characterized by a highly conserved C-terminal domain (EYA domain, ED) in which the tyrosine phosphatase activity resides. The N-terminal domain (NTD) of EYA is poorly conserved and possesses transactivation potential [2]. Although several reports have suggested the functional redundancy among EYA homologs, there is considerable evidence for the distinct functions of each EYA family member, particularly in cancers [2–3]. There is growing evidence that either gain-of-function or loss-of-function abnormalities of EYA members are implicated in various malignancies, arguing for context-specific roles for EYA dysregulation [4–17]. Of note, high EYA2 expression is significantly associated with aggressive phenotype and poor outcome in neuroblastoma (J.N. Hansen et al., unpublished data). Therefore, a systematic analysis of all EYA members should provide better understanding of the role of EYA in specific cancers, including neuroblastoma.

EYA1 exists in both the nucleus and cytoplasm and it has been recently shown that the role of aberrant EYA1 activity in cancer is likely to be related to its nuclear functions. Nuclear EYA1 is believed to be engaged in transcriptional regulation by association with transcriptional factors, as well as in the DNA damage response by dephosphorylating histones. These nuclear activities of EYA1 may mediate cancer cell proliferation, metastasis and chemoresistance [5–8]. Our analysis of EYA1 subcellular localization in neuroblastomas suggested an association between nuclear localization and high-risk tumors (Figures 2–3), although this small sample size did not permit attainment of statistical significance. Importantly, our results demonstrated a physical interaction between EYA1 and nuclear MYCN, a classical oncogenic transcription factor and major driver of neuroblastoma tumorigenesis, in both transfected human embryonic kidney cells and native neuroblastoma cells. Since MYCN shifts from having a predominately nuclear localization to cytoplasmic expression during neuronal differentiation[25], this suggests that especially high nuclear EYA1 protein expression may also be reflective of more primitive, and therefore proliferative, neuronal precursor state. It has been well-documented that neuroblastoma cells that lack amplified *MYCN* generally express MYC rather than MYCN [26, 27]. The physical interaction between EYA1 and MYC has been recently demonstrated in mouse nephron progenitors [22]. Our study extends the previous findings to human neuroblastoma cells and shows the association of nuclear EYA1 with nuclear MYCN in both human neuroblastoma cell lines (Figure 4b) and primary neuroblastoma tumors (Figure 4c and 4d). Furthermore, as high nuclear MYCN levels are closely related to advanced stage neuroblastomas, we propose that the correlation between high-level nuclear MYCN and nuclear localization of EYA1 would mainly occur in advanced stage neuroblastomas. These observations provide insights into the mechanistic basis of the seemingly paradoxical relationship between total EYA1 versus nuclear EYA1 levels and disease stage of neuroblastomas. This raises both the possibility of therapeutic modulation of EYA1 subcellular localization and the question of the mechanism by which nuclear translocation of EYA1 and EYA1-MYCN association are regulated during normal development and oncogenesis. Based on recent findings that EYA1 activity is regulated by post-translational modifications [23, 28], further studies on the function and (dys)regulation of EYA1 in neuroblastoma and other malignancies are critical to the potential exploration of EYA1 as a target for cancer therapy and drug development.

## Conclusions

Modulation of expression and subcellular localization of EYA1 in neural crest cells may provide novel therapeutic strategies for neuroblastoma.

## Figures and Tables

**Table 1:** EYA1 protein expression, EYA1 subcellular localization and prognostic factors.

	EYA1 protein expression			subcellular localization of high EYA1 tumors		

	Neg/low	High	P-value^a^	Cytoplasmic	Nuclear	P-value^a^
*Age at diagnosis*						
≤18 months	13	26	0.3131	11	16	1.000
>18 months	12	14		2	8	
*INSS stage*						
1, 2, 3, 4S	12	29	0.1154	10	18	0.3220
4	12	12		7	6	
*Histology*						
Favorable	7	11	1.0000	2	9	0.3615
Unfavorable	8	10		5	6	
*MKI*						
Low/Intermediate	13	20	0.6743	8	13	1.000
High	3	3		1	2	

*Abbreviations:* INSS: International Neuroblastoma Staging System; MKI: Mitosis-Karyorrhexis index;

a Two-tailed Fisher’s exact test.
